# Cryo-EM Pipeline for Actin Filament End Structures

**DOI:** 10.21769/BioProtoc.5794

**Published:** 2026-09-05

**Authors:** Nicholas J. Palmer, Roberto Dominguez

**Affiliations:** Department of Physiology and Biochemistry, Biophysics and Chemical Biology Graduate Group, University of Pennsylvania, Perelman School of Medicine, Philadelphia, PA, USA

**Keywords:** Cryo-EM, Actin filament, Filament ends, Machine learning, Vitrification, Protein structure, Cytoskeleton

## Abstract

Actin filaments undergo dynamic growth and disassembly at their ends, regulated by many actin-binding proteins. However, structural analysis of filament end dynamics has been challenging due to the low abundance of filament ends in cryo-electron microscopy (cryo-EM) micrographs, their intrinsic polymorphisms, and the diversity and flexibility of end-binding proteins. Here, we describe a standardized cryo-EM protocol for determining actin filament end structures. First, short actin filaments are generated either biochemically using capping or severing proteins or mechanically through shearing. Filaments are then vitrified under conditions optimized for each specific end-binding protein. We describe data collection parameters using a 300 kV Titan Krios G3i microscope, including optimized grid preparation and imaging settings. Finally, we present a data processing pipeline for filament end structure determination based on machine learning–based particle picking, masking, and sorting strategies. This protocol has enabled the determination of multiple high-resolution structures of free, capped, elongating, and depolymerizing actin filament ends, and we further discuss considerations for extending this approach to other end-binding proteins.

Key features

• Generation of short actin filaments for cryo-EM using capping proteins, severing proteins, or mechanical shearing.

• Integrated data collection and image processing workflow for filament end structure determination.

• Common challenges, solutions, and experimental considerations for diverse actin filament end-binding complexes.

## Graphical overview


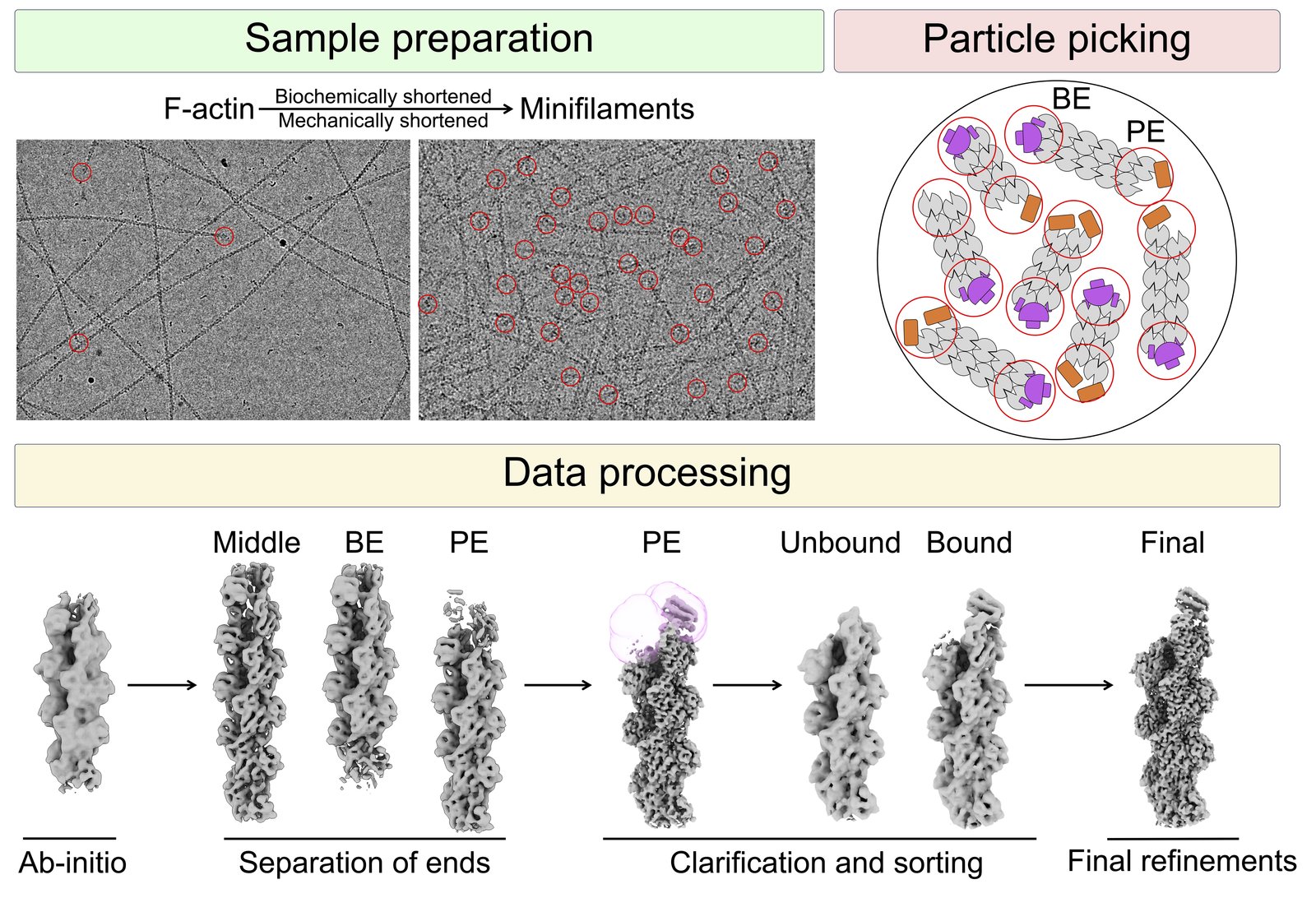


## Background

Actin is a highly conserved and abundant protein that participates in numerous protein–protein interactions and plays crucial roles in cell motility, cytokinesis, and intracellular trafficking [1]. Central to actin’s functions is its ability to transition between monomeric (G-actin) and filamentous (F-actin) states in a highly dynamic and regulated manner, with an approximately 50:50 distribution between these two states in cells. F-actin is a polar filament with fast- and slow-growing ends, known as the barbed and pointed ends, respectively [2]. In cells, filament end dynamics are tightly regulated by a variety of barbed- and pointed-end binding proteins that control polymerization and depolymerization [1,3]; dysregulation of these processes is implicated in multiple human diseases [4,5].

High-resolution structures of filament ends are essential for understanding actin dynamics at the molecular level, but obtaining such structures has proven challenging. Cryo-electron microscopy (cryo-EM), the primary technique used, relies on averaging thousands of particles to reconstruct three-dimensional maps of proteins and complexes [6]. However, actin filaments can extend over micrometers, often spanning entire micrographs, resulting in very few filament ends per image. To overcome this limitation, filaments can be shortened either biochemically, using proteins that cap, slow filament growth, or sever filaments, or mechanically through shearing. Additional considerations include the activity of the end-binding protein under study, such as whether it interacts stably or transiently with filament ends or alters the polymerization and depolymerization rates. A further challenge is particle picking, as classical methods such as blob- or template-based approaches often fail to distinguish filament ends from filament middles. Machine learning–based picking methods implemented in software such as Topaz [7] and crYOLO [8] can be trained to address this limitation.

Here, we describe a step-by-step workflow for determining cryo-EM structures of actin filament ends using cryoSPARC [9,10] and highlight recently published examples in which this workflow has been applied successfully [11–15]. This framework may also be applicable to other classes of filamentous proteins and end-binding proteins.

## Materials and reagents

**Biological materials**

1. Rabbit alpha skeletal muscle actin (UniProt P68135), purified in our laboratory from rabbit muscle using an established protocol [16]; alternatively, actin can be purified from commercially available muscle acetone powder from sources such as Cytoskeleton (catalog number: SKU: AKL99) or Pel-Freez Biologicals (catalog number: 41995-2)

*Note: Actin preparations should be made fresh and stored in G-buffer (see Recipes) on ice or at 4 °C. A fresh preparation can last between 1 and 3 weeks in these conditions before a new preparation should be made.*

2. Capping protein (CP) (UniProt: P52907 and P47756-2), purified as a heterodimer as described previously [11]

3. Cyclase associated protein-1 construct (UniProt: Q01518), purified as described previously [15]

**Reagents**

1. HEPES (Gold Biotechnology Inc, catalog number: H-400-1)

2. Potassium chloride (KCl) (Santa Cruz Biotechnology, catalog number: sc-203207A)

3. Ethylene glycol tetraacetic acid (EGTA) (Gold Biotechnology Inc, catalog number: E-217-100)

4. Magnesium chloride (MgCl_2_) (Fisher Scientific Company, catalog number: 442611500GM)

5. Dithiothreitol (DTT) (Gold Biotechnology Inc, catalog number: DTT100)

6. Adenosine 5′-triphosphate (ATP) (VWR International LLC, catalog number: 77877-060)

7. Tris base (Santa Cruz Biotechnology, catalog number: sc-3715C)

8. Calcium chloride dihydrate (CaCl_2_) (LabChem, catalog number: LC127251)

*Note: It is important to store DTT and ATP as frozen aliquots at -20 °C and use freshly thawed aliquots for sample preparation. All buffers containing DTT and ATP should be made fresh.*

**Solutions**

1. G-buffer in G-buffer (see Recipes)

2. Capped filaments in F-buffer (see Recipes)

3. F-actin in F-buffer (see Recipes)

**Recipes**

**1. G-buffer**

**Table:** 

Reagent	Final concentration	Quantity or volume
1 M Tris, pH 8.0	5 mM	2.5 μL
20 mM CaCl_2_	0.2 mM	5 μL
20 mM ATP	0.2 mM	5 μL
20 mM DTT	0.2 mM	5 μL
H_2_O	n/a	482.5 μL
Total	n/a	500 μL

**2. Capped filaments in F-buffer**

**Table:** 

Reagent	Final concentration	Quantity or volume
1 M HEPES pH 7.5	20 mM	2 μL
2.5 M KCl	50 mM	2 μL
0.1 M EGTA	1 mM	1 μL
0.1 M MgCl_2_	1 mM	1 μL
0.1 M ATP	1 mM	1 μL
0.1 M DTT	1 mM	1 μL
H_2_O	n/a	42 μL
29.4 μM capping protein (CP)	5 μM	17 μL
75.8 μM G-actin	25 μM	33 μL
Total	n/a	100 μL

**3. F-actin in F-buffer**

**Table:** 

Reagent	Final concentration	Quantity or volume
1 M HEPES pH 7.5	20 mM	2 μL
2.5 M KCl	50 mM	2 μL
0.1 M EGTA	1 mM	1 μL
0.1 M MgCl_2_	1 mM	1 μL
0.1 M ATP	1 mM	1 μL
0.1 M DTT	1 mM	1 μL
H_2_O	n/a	72 μL
75.8 μM G-actin	15 μM	20 μL
Total	n/a	100 μL

**Laboratory supplies**

1. Standard Vitrobot filter paper grade 595 (Ted Pella, Inc, catalog number: 47000-100)

2. Quantifoil R1.2/1.3 300 mesh, copper grids (Quantifoil, https://www.quantifoil.com/products/quantifoil/quantifoil-circular-holes)

3. Autogrid rings and C-clips (Electron Microscopy Sciences, catalog number: 71167-97)

## Equipment

1. PELCO easiGlow Glow Discharge (PELCO, Ted Pella, Inc, catalog number: 91000S)

2. Ultrasonic Cleaner (Fisher Scientific, catalog number: FS20D)

3. Slide-Clamp Tweezers Assembly for FEI Vitrobot Mark IV-I (Ted Pella, Inc, catalog number: 47000-500)

4. Vitrobot Mark IV (Thermo Fisher)

5. Vitrobot dewar with metal parts (Ted Pella, Inc, catalog number: 47000-705)

6. Cryo grid boxes (Sub-Angstrom, catalog number: GBV01)

7. Krios G3i Cryo-Transmission Electron Microscope, 300 kV (Thermo Fisher)

8. K3-GIF Direct Electron Detector equipped with BioQuantum K3 imaging filter (Gatan Inc., AMETEK)

9. Customized 8 GPU Cryo-EM workstation (Single Particle)

## Software and datasets

1. CryoSPARC (Structura Biotechnology, v4.7, dependencies and prerequisites can be found at https://guide.cryosparc.com/)

2. UCSF ChimeraX (https://www.cgl.ucsf.edu/chimerax, v1.10.1)

3. Topaz (https://github.com/3dem/topaz, v0.3.0, dependencies can be found in the GitHub repository)

4. EPU (Thermo Fisher, v3.12)

## Procedure

**A. Preparation of short actin filaments**

Short actin filaments are typically prepared prior to sample vitrification, and their preparation is adjusted according to the biochemical activity of the end-binding protein under investigation. For proteins that cap (e.g., CP, tropomodulin) or sever (e.g., gelsolin, INF2) filaments, the protein may be added either during F-actin assembly or before blotting, respectively. At sufficiently high concentrations, capping and severing proteins are often sufficient to limit elongation and maintain filaments short.

It is also important to consider the affinity and activity of the end-binding protein under investigation. For instance, CP binds with high affinity to the barbed end [17], effectively blocking subunit exchange, whereas tropomodulin binds weakly and transiently to the pointed end [11,18], allowing slow subunit exchange. In the example described here, we used a construct of cyclase-associated protein (CAP), which binds transiently to the pointed end and adopts multiple conformations. To generate short filaments, in this case, actin filaments were polymerized in the presence of high concentrations of CP, and CAP was added prior to blotting since it can act as a pointed end depolymerase.

**A1. Preparation of short filaments using CP**

1. Prepare reagents:

a. 2 μL of HEPES pH 7.5 buffer at 1,000 mM (final concentration = 20 mM)

b. 2 μL of KCl at 2,500 mM (final concentration = 50 mM)

c. 1 μL of EGTA at 100 mM (final concentration = 1 mM)

d. 1 μL of MgCl_2_ at 100 mM (final concentration = 1 mM)

e. 1 μL of ATP at 100 mM (final concentration = 1 mM)

f. 1 μL of DTT at 100 mM (final concentration = 1 mM)

g. 42 μL of deionized H_2_O (diH_2_O)

2. Add CP to a final concentration of 5 μM and mix thoroughly but gently.

3. Add G-actin to a final concentration of 25 μM and mix thoroughly but gently. Adjust with ddH_2_O so that the final volume is 100 μL after addition of actin.

4. Incubate the mixture at room temperature for at least 1 h prior to blotting.

5. Proceed to section B1 for the vitrification protocol.

**A2. Preparation of F-actin for shearing**

If the end-binding protein under investigation does not cap or sever filaments, mechanical shearing using either a sonicator or syringe may be the only available option for generating shorter filaments. However, shearing is generally less effective than capping or severing, as filaments tend to reanneal rapidly; therefore, thorough shearing followed by immediate freezing is important. Importantly, for shearing, the actin concentration should be lower than that used in biochemical approaches, as higher concentrations promote faster reannealing.

1. Prepare reagents:

a. 2 μL of HEPES pH 7.5 buffer at 1,000 mM (final concentration = 20 mM)

b. 2 μL of KCl at 2,500 mM (final concentration = 50 mM)

c. 1 μL of EGTA at 100 mM (final concentration = 1 mM)

d. 1 μL of MgCl_2_ at 100 mM (final concentration = 1 mM)

e. 1 μL of ATP at 100 mM (final concentration = 1 mM)

f. 1 μL of DTT at 100 mM (final concentration = 1 mM)

g. 72 μL of diH_2_O

2. Add G-actin to a final concentration of 15 μM and mix thoroughly. The total volume should now be 100 μL.

3. Incubate at room temperature for at least 1 h.

**B. Vitrification**

**B1. Vitrification of biochemically shortened filaments**

If filaments have been generated through the addition of CP, the sample can be treated as a standard cryo-EM specimen and blotted under routine conditions. In the present case study, CAP was added 5 min prior to blotting to filaments generated in the presence of CP.

1. Turn on the Vitrobot Mark IV. Water should be injected into the Vitrobot humidifier if the chamber is empty.

2. Set temperature to 4 °C, humidity to 100%, and turn on humidity control. Close the Vitrobot door and allow the system to equilibrate, which should take around 10–20 min.

3. Set blotting time and force to the desired values (typical settings: blotting time, 2.5–3 s; force, 3).

*Note: It is recommended to screen a range of blotting conditions when establishing protocols for a new sample.*

4. Assemble the Vitrobot foam dewar by placing it in the grid box holder, ethane container, and spider.

5. Cool the assembled dewar with liquid nitrogen, ensuring that liquid nitrogen fills the outer reservoir.

6. Once sufficiently cooled, condense ethane into the ethane container. This chamber is where the sample is rapidly vitrified. The nitrogen outer layer is used to initially cool the system and to store vitrified grids throughout the procedure.

7. Glow-discharge Quantifoil R1.2/1.3 grids using a PELCO easiGlow or equivalent system. The settings used in this protocol for glow discharging were a 0.38 mBar pressure with a current of 15 mA for 1 min of exposure time.

**Critical:** Ensure that the carbon side of the grids is facing upward during glow discharge.

8. Load Vitrobot filter paper onto the blotting pads.

9. Remove the spider from the Vitrobot dewar prior to blotting, making sure that the ethane and nitrogen are clean and free of ice contamination.

10. Add CAP to the filaments prepared in section A1 5 min prior to freezing and mix well. The final concentration of each component is 20 μM CAP, 4.8 μM CP, and 24 μM actin.

*Note: The final concentration of all components will vary depending on the concentration of the protein you’re adding. In general, the final concentration of F-actin should be between 20 and 35 μM, which allows for ample free ends with less particle crowding. Testing ranges of concentrations for each new protein is advisable. The final concentration of your protein of interest should also be high, which increases occupancy at the end it binds and helps prevent filament reannealing.*

11. Pick up a glow-discharged grid on its outer circumference using the Vitrobot tweezers and lock the forceps to hold the grid in place. Load the forceps onto the Vitrobot plunging rod and pull them into the freezing chamber. Orient the grid so that the carbon side faces the blotting pad.

12. Apply 3 μL of the final minifilament sample to the carbon side of the grid, blot using set time and force, and plunge-freeze into liquid ethane using the Vitrobot system.

13. Transfer frozen grids to labeled grid boxes and store using Falcon tubes submerged in liquid nitrogen dewars until it is time for screening and/or data collection. Prior to data collection, grids must be “clipped” into autogrid rings for use with the Titan Krios autoloader.

14. Refer to the Troubleshooting section if issues with preferred orientation are encountered during data processing, as this can occur with some end-binding proteins. Additives during vitrification may be able to ameliorate these issues.

**B2. Vitrification of mechanically sheared F-actin**

See **Video 1** for a full demonstration of the filament shearing and blotting process.

**Video 1.**
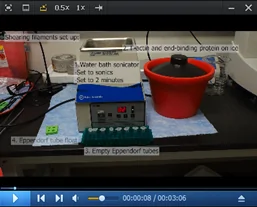
Vitrification of sheared actin filaments

1. Set up a water bath sonicator by filling it with water, selecting the sonication mode, and setting the cycle to 2 min.

2. Prepare two pipettes: one set to aspirate 3-μL volumes and another set to the volume required for the addition of the end-binding protein to the sheared F-actin sample.

3. Repeat steps B1.1–9 prior to shearing the filaments.

4. Place a glow-discharged Quantifoil R1.2/1.3 grid in position for blotting in the Vitrobot system. Have it set waiting in the position ready to accept the sample.

**Pause point:** Before proceeding with filament shearing and blotting, ensure that all equipment is set up and ready for immediate use.

5. Add 10 μL of F-actin in F-buffer to an Eppendorf tube.

6. Place the Eppendorf tube in a floating holder within the water bath and initiate the sonication cycle.

7. During the sonication cycle, aspirate the end-binding protein in a pipette, ensuring readiness for immediate addition to the sheared F-actin upon completion of sonication. The final concentrations of the components at this step are 15 μM CAP and 13.6 μM F-actin.

*Note: The amount of end-binding protein added depends on its affinity, desired final concentration, and the concentration of F-actin. For filament shearing, a final F-actin concentration between 10 and 20 μM is optimal to limit filament reannealing. Higher concentrations of end-binding proteins also help prevent reannealing, since most end-binding proteins inhibit this process by blocking one of the filament ends.*

8. Immediately upon completion of the sonication cycle, remove the Eppendorf tube from the water bath and add the end-binding protein to the sheared filaments, mixing thoroughly but briefly.

9. Immediately apply the sample to the grid in the Vitrobot and proceed with blotting and plunge-freezing.

10. Repeat steps B2.4–9 for each grid.

11. Transfer the vitrified grids to labeled grid boxes and store them in Falcon tubes submerged in liquid nitrogen dewars for long-term storage until screening and/or data collection. Prior to data collection, grids must be “clipped” into autogrid rings for use with the Krios autoloader.

12. Refer to the Troubleshooting section if issues with preferred orientation are encountered during data processing, as this can sometimes occur with end-binding particles. Additives during vitrification may be able to ameliorate these issues.

**C. Data collection considerations**

It is advisable to first consult the microscope operators regarding the optimal setup for the specific project and instrumentation available. In most standard cryo-EM projects, 50 or more particles per micrograph can usually be obtained. However, even under optimal conditions, achieving this number of filament ends may be difficult. In practice, 10–30 filament ends per micrograph is acceptable. It is also important to consider that filament ends are divided between barbed and pointed ends, effectively reducing the number of particles of the desired end type by half.

The number of movies to be collected often depends on the availability and cost of microscope time, as well as storage capacity. Most filament end projects in our laboratory have required at least 20,000 movies, collected at 81,000× magnification, to achieve complete and high-resolution structures. This requirement may vary, as some end-binding proteins exhibit multiple structural states or lower occupancy, resulting in fewer particles per state and necessitating multiple data collection sessions. In many cases, the need for additional data collection can only be fully assessed after processing an initial dataset.

On our Titan Krios, typical data collection parameters include super-resolution acquisition mode, reaching a pixel size of 0.54 Å, corresponding to 81,000× magnification, an electron dose of approximately 50 e-/Å^2^, and defocus values between -0.5 and -2.5 μm. A pixel size of 0.54 Å at super-resolution (corresponding to a physical pixel size of 1.08 Å) has the potential to achieve high-resolution structures with a Nyquist limit of 1.08 Å. In practice, end-structures will not achieve this limit due to other factors such as particle count and polydispersity. The maximum resolutions we have achieved so far are approximately 2.6 Å for CP, a protein that binds the BE tightly and mostly adopts a single conformation [14,15]. However, at this pixel size, the magnification is at an optimal point, allowing two exposures per hole using R1.2/1.3 grids and, depending on protein concentration, more than 10 ends per micrograph. Other magnifications/pixel sizes can and should be tested while screening grids and balancing the pixel size, number of ends visible, and number of shots per hole for optimal data collection.

**D. Generating a mask for cryo-EM classification**

Creating appropriate masks is key for several steps during the following data processing section, so a more detailed protocol for mask generation will first be described here. The method of mask generation used in this protocol requires having an approximate PDB model based on prior structural information or generated by prediction software like AlphaFold [19]. If this is not possible, other methods described in the cryoSPARC guide can be used. For this project, a crystal structure of the helical-folded domain (HFD) of N-CAP bound to G-actin had already been solved by crystallography (PDB code: 6RSW) and was used to create the initial masks [20].

1. Start UCSF ChimeraX [21] on your local workstation and open the map you want to make a mask for and the PDB file that will be used to create it.

2. Place the PDB structure near its suspected position in the map by using the *Rotate and Move Model* commands under the *Right Mouse* tab. Once placed, use the *Fit* command under the map tab, which will place the structure into the map using rigid-body local optimization.

**Pause point:** Since we are initially using an approximate model, it may not fit accurately. Therefore, make sure that the fitted model covers most of the area you want to mask before proceeding. During processing, it is appropriate to revise the initial model based on non-finalized data from the processing steps below.

3. Select the portion of the PDB model that contains the area you want to mask using ChimeraX’s selection command.

4. Once all atoms are selected, use the following command to generate a volume from the model selected.

>molmap sel 16 onGrid #1

This command will generate a map from the selection on the coordinate system of volume ID #1 at a resolution of 16 Å. If the volume does not have the ID of #1, use the ID listed for it as seen in the *Models* panel.

5. In the *volume viewer* panel, move the slider so that the generated map is continuous and has no floating dust; note the threshold that is in the **Level** box above.

6. Check again to make sure the generated map is in the desired area and save it as an .mrc file on your workstation.

7. Transfer this .mrc file to the system running cryoSPARC, either through an SCP transfer, dragging the file into cryoSPARC in a web browser, or by your preferred method of file transfer.

8. In your cryoSPARC workspace, import the volume using the ***Import 3D Volume*** job:

Volume data path: /Path/to/volume/*.mrc

Type of volume being imported: map

9. Now that the volume is imported into the cryoSPARC project, create a ***Volume Tools*** job using the imported volume:

Type of input volume: map

Type of output volume: mask

Threshold: varies (based on the noted threshold from step D5)

Dilation radius (pix): 6–12 (the higher the dilation radius, the more the volume will encompass)

Soft padding width (pix): 12–24 (varies, but a larger value is recommended to prevent edge artifacts)

*Note: The dilation radius and soft padding width can vary significantly during mask generation. It is recommended to try tighter or wider masks if downstream 3D classification is not effectively separating classes.*

10. The mask is now prepared for use in 3D classification or local refinement jobs.

**E. Data processing**

**Figure 1.**
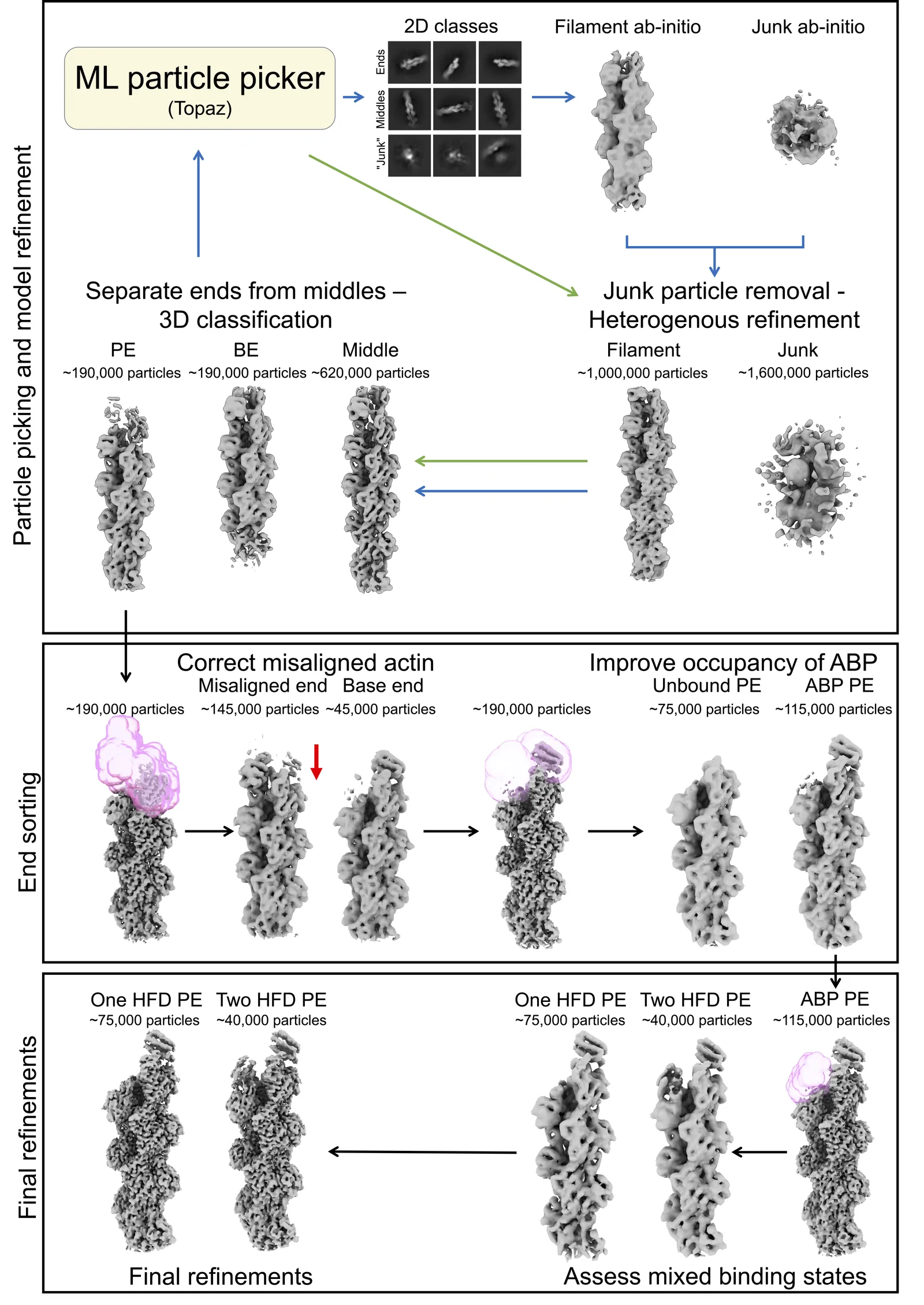
Workflow for the determination of actin filament end structures. (Top) A machine-learning particle picker, such as Topaz [7] or crYOLO [8], is trained using manually selected filament ends and used to identify initial particles. These particles are then filtered to retain filament ends and used to further improve the machine-learning particle picker. This process is iterated without generating new ab initio volumes until additional filament ends are reliably identified. (Middle) Filament-end particles are then sorted, beginning with realignment of filament ends, followed by separation into unoccupied and occupied ends. (Bottom) Mixed binding states are resolved. Further refinement yields final volumes suitable for model building and refinement. Approximate particle counts for each step are given, but can vary depending on the ML picking model, number of particles per micrograph, and topaz particle threshold.

For the example here, particle picking was performed using Topaz [7], and data processing was carried out in cryoSPARC [9,10]. Similar workflows can be implemented in other software packages, including RELION [22]. The data here was collected at a 0.54 Å pixel size with an 81,000× magnification, 300 kV accelerating voltage, and a total exposure dose of 49.6 e-/Å^2^. The overall processing workflow is summarized in Figure 1.

1. Import movies into cryoSPARC using the ***Import Movies*** job:

Movies data path: /Path/to/movies/*.tiff

Gain reference path: /Path/to/gainref/gain.mrc

Raw pixel size (Å): 0.54

Accelerating voltage (kV): 300

Spherical aberration (mm): 2.7

Total exposure dose (e-/Å^2^): 49.6

All other parameters: default settings

*Note: Raw pixel size, accelerating voltage, spherical aberration, and total dose depend on the specific microscope and magnification used. The values provided here correspond to the case study used.*

2. Perform motion correction on the imported movies using the ***Patch Motion Correction*** job:

Save results in 16-bit floating point: on (saves 50% on disk space with effectively no resolution loss)

Output F-crop factor: 1/2

Number of GPUs to parallelize: variable (increased number of GPUs reduces runtime)

All other parameters: default settings

*Note: Fourier cropping during motion correction is often advised as it reduces the pixel size, increasing the speed of upstream processing and decreasing the storage space requirement of extracted particles. This does, however, limit the achievable resolution according to the Nyquist limit. If this limit is achieved during processing, the particles themselves can be uncropped during a* ***Reference-Based Motion Correction*** *job at the final step. In this example, the output F-crop factor was set to 1/2, reducing the 0.54 Å pixel size to 1.08 Å and limiting the maximum achievable resolution to 2.16 Å. The final resolutions of the end structures reached only ~3.0 Å, and so the particles never needed to be uncropped later during* ***Reference-Based Motion Correction***.

3. Estimate the contrast transfer function (CTF) using the ***Patch CTF*** job:

Number of GPUs to parallelize: variable (increased number of GPUs reduces runtime)

All other parameters: default settings

**Figure 2.**
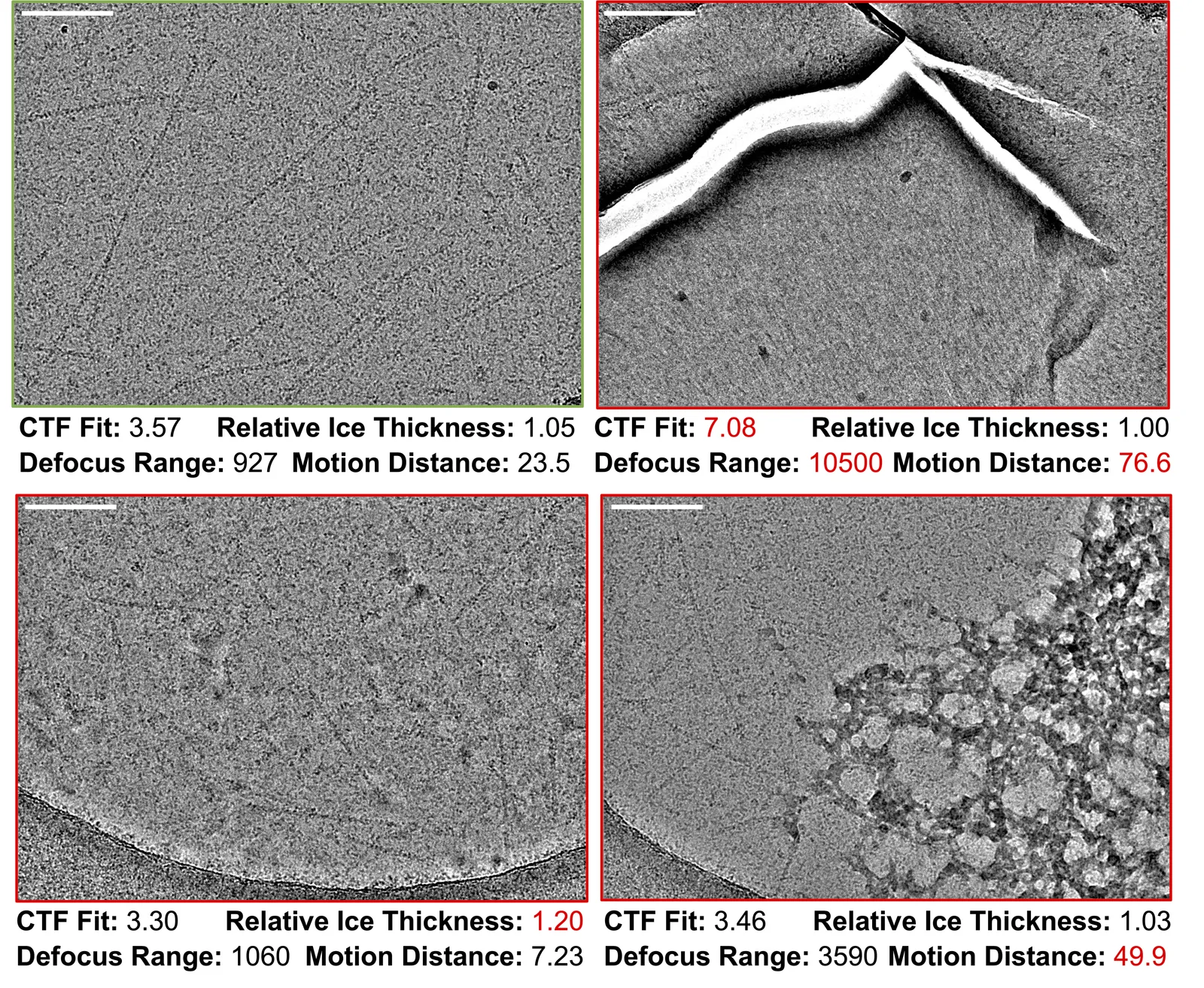
Example curated micrographs. Example micrographs curated using the *Manually Curate Exposures* job, with an accepted micrograph (contoured green), and several rejected micrographs (contoured red). CTF fit, relative ice thickness, defocus range, and motion distance for each micrograph are provided below, and parameters outside of the threshold used by this example are highlighted in red. Scale bars, 100 nm.

4. Curate exposures using the ***Manually Curate Exposures*** job. This interactive interface allows filtering based on multiple parameters that may vary between datasets. Commonly excluded outliers include micrographs with aberrant average defocus, poor CTF fit resolution, abnormal average intensity, excessive ice thickness, or high full-frame motion. Additional manual inspection is recommended to remove micrographs with contamination or damaged holes. Examples of accepted and rejected micrographs curated during selection are shown in Figure 2.

***Manually Curate Exposures*** job cutoffs used in this example:

CTF fit resolution (Å): 1–6 (outliers likely have poor CTF fitting)

Average intensity: (-123)-116 (outliers are likely contaminated or damaged)

Relative ice thickness: 0.98–1.08 (outliers are likely crystalline ice)

Total full-frame motion distance (pixels): 0–32 (outliers are broken holes or have heavy contamination)

Defocus range (Å): 30–2653 (a larger range here could indicate contamination)

**Caution:** These values can vary for different data sets collected with different parameters or on different microscopes.

**Figure 3.**
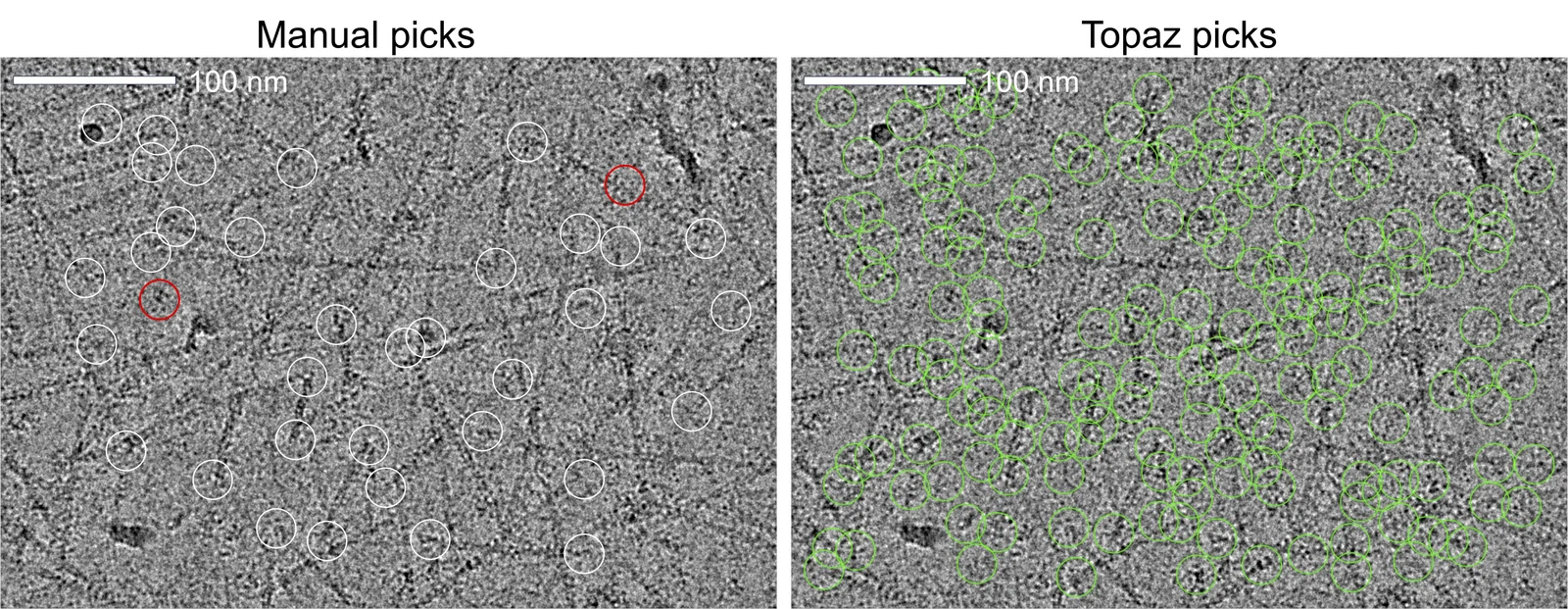
Particle picking. (Left) Manually picked filament ends (white circles) used for initial machine-learning model training. Two circles in red highlight a junk particle and a mid-filament particle, which should not be picked during manual selection. (Right) Particles picked by Topaz (green circles). Note that Topaz identifies a larger number of filament ends but also picks mid-filament and “junk” particles.

5. From the curated micrographs, manually select filament ends using the ***Manual Picker*** job. This interface enables visual inspection and particle selection. To start, select more than 1,000 filament ends across as many micrographs as needed by left-clicking within the interface (see example in **Figure 3**, left).

*Note: The* ***Manual Picker*** *job allows sorting by average defocus; higher defocus micrographs can improve visual identification of filament ends. If filament ends remain difficult to identify, increasing the low-pass filter using the slider in the interface may improve visualization.*

6. Once ~1,000 particles have been manually selected, they can be used to train a Topaz particle-picking model. Using the ***Topaz Train*** job, input both the micrographs and particles from the ***Manual Picker*** job. Importantly, do not use all collected exposures for training; only include exposures containing manually picked particles. The estimated particle diameter is based on the diameter perpendicular to the longitudinal actin filament axis plus any increment accounting for the end-bound protein. A good estimate can be obtained from a premade model and refined through tests with different Topaz models.

**Caution:** It is important to only include exposures containing manually picked particles at this point. Including micrographs that contain unselected ends will poison the ML model with false negatives. For the same reason, only ends should be selected rather than middles, which will help train the ML model to select a higher ratio of ends to middles in a subsequent ***Topaz Train*** job.

Path to Topaz executable: /Path/to/topaz/bin/topaz

Number of parallel processes: variable

Downsampling mode: auto

Estimated particle diameter (Å): 100 (may vary depending on the end-binding protein)

Expected number of particles: 50 (anecdotally, slight overestimation may improve model performance)

Number of epochs: 10–30 (increase if the model appears to have room to improve)

Number of CPUs: variable

All other parameters: default settings

7. Input the Topaz model generated from the ***Topaz Train*** job, together with all curated micrographs from the ***Manually Curate Exposures*** job from step D4, into a ***Topaz Extract*** job. This step uses the trained model to identify particles across all selected exposures (see example in **Figure 3**, right).

Path to Topaz executable: /Path/to/topaz/bin/topaz

Downsampling mode: auto

Estimated particle diameter (Å): 100 (may vary depending on the end-binding protein)

Number of parallel processes: variable

Particle threshold: -1 (increase or decrease by increments of 1 if too many or too few particles are being picked)

Number of CPUs: variable

All other parameters: default settings

*Note: Topaz may run significantly faster when processing only a portion of the dataset. On our system (see Equipment section), we typically process no more than 5,000 exposures per* ***Topaz Train*** *or* ***Topaz Extract*** *job. To process an entire dataset, micrographs can be divided into smaller groups using the* ***Exposure Sets Tool***, *followed by separate* ***Topaz Extract*** *jobs for each subset. This tool can also be used to randomize datasets, ensuring that the* ***Topaz Train*** *job includes micrographs sampled from different regions of the grid. Once all particle locations have been picked by each* ***Topaz Extract*** *job, you can extract all particles at the same time by inputting each output particle stack into an* ***Extract From Micrographs (GPU)*** *job using all curated micrographs.*

8. Extract the Topaz picked particles from their micrographs using the ***Extract From Micrographs (GPU)*** job. For actin end particles, the box size should be large enough to encompass five or more actin subunits with the filament end at or near the center. This leaves sufficient room for downstream 3D classifications to obtain distinct barbed and pointed end classes based on the absence of actin protomers. At this early stage, it is possible to further Fourier crop during extraction from micrograph jobs. This plays the same role as during motion correction, reducing their file size and increasing the processing speed while simultaneously reducing the maximal achievable resolution. Fourier cropping by 4–8 at this stage will not make the distinction of ends for middles difficult, but will significantly speed up processing. For this example, a box size of 412 was used for the overall box size, which incorporated enough actin subunits and left plenty of room for their absence at each end.

Number of GPUs to parallelize (0 for CPU-only): Variable

Extraction box size (pix): 412 (depending on particle and pixel size)

Save results in 16-bit floating point: on (saves 50% on disk space with effectively no resolution loss)

Fourier crop to box size (pix): 52 (using 1/8^th^–1/4^th^ the extraction box size is recommended at this stage)

All other parameters: default settings

9. Generate 2D classes from the extracted particles using the ***2D Classification (GPU)*** job. Manually inspect 2D classes to assess the ability of the Topaz model for picking filament ends. Only a small number of end classes may appear here, since filament end particles may also be classified into middle filament 2D classes.

Number of 2D classes: 75 (depending on the number of input particles)

Recenter 2D classes: off (if left on, end particles will more often be filtered into middle filament classes)

Number of GPUs to parallelize: variable

All other parameters: default settings

10. From the generated 2D classes, create two ***Select 2D Classes*** jobs. For the first job, select any 2D classes resembling filaments; this will include both filament ends and mid-filament classes. For the second job, select a few 2D classes that are clearly “junk.” Examples of end, middle, and junk 2D classes are shown in **Figure 1** (top panel). It is important to note that junk classes may contain good particles with rare poses; however, these classes will only be used to generate initial filament models for subsequent heterogeneous refinement jobs. In this protocol, 2D classification is not used to select particles for final refinements.

11. Input the particles from each ***Select 2D Classes*** job into two separate ***Ab-Initio Reconstruction*** jobs. The volumes generated will be used as the initial filament volume and junk volume.

Number of Ab-initio classes: 1–3

All other parameters: default settings

12. Use the filament and junk ab-initio volumes and all particles from the previous 2D classification job in step D9 in a ***Heterogeneous Refinement*** job. Do not use the particles from the ***Select 2D Classes*** job.

All other parameters: default settings

*Note: Multiple rounds of heterogenous refinement may be required to remove all “junk” particles. If junk particles are suspected to remain within a filament class, clone the old* ***Heterogeneous Refinement*** *job and use only the particles that were sorted into the filament class in a new* ***Heterogenous Refinement*** *job.*

13. Once junk particles are removed, use the particles from the filament class in a ***3D Classification*** job. No other inputs will be necessary for this 3D classification. The result of this ***3D Classification*** should be one class of actin filament barbed ends, one class of pointed ends, and multiple mid-filament classes. If this does not work, refer to the Troubleshooting section.

Number of classes: 5

Filter resolution (Å): 6

All other parameters: default settings

*Note: 3D classifications will generate a series of volumes that are filtered to approximately 6 Å, which should be the resolution where heterogeneity is expected. Distinguishing ends from middles often does not need high-resolution reconstructions; however, any class can be used in a subsequent non-uniform refinement job to assess higher-resolution features if they are anticipated.*

14. We can now use the filtered particles to train a better Topaz model. Use both the barbed and pointed end particles from the ***3D classification*** job as inputs for a new ***Topaz Train*** job. Use the maximum number of micrographs your system can handle for this job.

Path to Topaz executable: /Path/to/topaz/bin/topaz

Number of parallel processes: variable

Downsampling mode: auto

Estimated particle diameter (Å): 100 (may vary depending on the end-binding protein)

Expected number of particles: 50 (anecdotally, slight overestimation may improve model performance)

Number of epochs: 10–30 (increase if the model appears to have room to improve)

Number of CPUs: variable

All other parameters: default settings

15. Repeat the ***Topaz Extract*** job using the new Topaz particle picking model and using all micrographs, followed by an ***Extract From Micrographs (GPU)*** job.

***Topaz Extract*** job:

Path to Topaz executable: /Path/to/topaz/bin/topaz

Downsampling mode: auto

Estimated particle diameter (Å): 100 (may vary depending on the end-binding protein)

Number of parallel processes: variable

Particle threshold: -1 (increase or decrease by increments of 1 if too many or too few particles are being picked)

Number of CPUs: variable

All other parameters: default settings

***Extract From Micrographs(GPU)*** job:

Number of GPUs to parallelize (0 for CPU-only): variable

Extraction box size (pix): 412 (depends on particle and pixel size)

Save results in 16-bit floating point: on (saves 50% on disk space with effectively no resolution loss)

Fourier crop to box size (pix): 52 (using 1/8^th^–1/4^th^ the extraction box size is recommended at this stage for speed)

All other parameters: default settings

16. Clone the original ***Heterogenous Refinement*** job from step D12, retaining its original volumes but using the new particles from the last ***Extract From Micrographs(GPU)*** job. This will remove the new junk particles picked by the new Topaz model.

17. Clone the original ***3D Classification*** job from step D13 using the filament particles from the new ***Heterogenous Refinement*** job. This 3D classification usually (but not always) results in more filament end particles. From here, proceed with the end class where you expect your end-binding protein to be present. In this case study, CAP binds the pointed end, so only the pointed end class will be used.

*Note: The* ***Remove Duplicate Particles*** *job can also be used with the results from the first and second rounds of* ***3D classification***, *which may increase the number of end particles. Often, the particles picked during the first round of Topaz do not completely overlap with those identified in the second round.*

18. Taking only the pointed end particles, re-extract them at full box size using an ***Extract From Micrographs (GPU)*** job.

Number of GPUs to parallelize (0 for CPU-only): variable

Extraction box size (pix): 412 (depends on particle and pixel size)

All other parameters: default settings

19. Input the extracted end-particles into a ***Non-uniform Refinement*** job. For the input volume, use the filament end volume generated in the ***3D classification*** from step D17.

Symmetry: C1 (unlike filament middles, filament ends will have no symmetry)

All other parameters: default settings

20. After non-uniform refinement is complete, filament ends may appear misaligned by one or more actin protomers. To correct this, generate a mask around the terminal subunits as depicted in **Figure 1** (middle panel). The mask should be broad and encompass all potentially misaligned subunits as well as the terminal actin subunits. Use this mask in a ***3D Classification*** job together with the particles from the previous ***Non-uniform Refinement*** job.

Number of classes: 5

Filter resolution (Å): 6

All other parameters: default settings

*Note: The protein of interest may become its own class during this* ***3D Classification*** *job. It is often preferable to focus only on aligning actin subunits at this stage and separate classes containing the protein of interest in subsequent jobs.*

21. The ***3D classification*** job should yield one or more filament-end classes in which the terminal actin subunits are misaligned relative to the center of the box. Using multiple ***Volume Alignment Tools*** jobs, recenter each particle class and corresponding volume so that the terminal actin subunit is positioned at the center of the box (**Figure 1**, middle panel). Using map-visualization software such as UCSF ChimeraX [21], verify that the ends of each shifted volume align correctly. Then, re-extract all shifted particles using another ***Extract From Micrographs (GPU)*** job, making sure to select *Recenter using aligned shifts*.

***Volume Alignment Tools*** job:

3D coordinates of new center (A or px): variable (shift the volume so that the tip of the terminal actin protomer is positioned at the center of the box)

All other parameters: default settings

***Extract from Micrographs (GPU)*** job:

Number of GPUs to parallelize (0 for CPU-only): variable

Extraction box size (pix): 412 (depends on particle and pixel size)

Save results in 16-bit floating point: on (saves 50% on disk space with effectively no resolution loss)

Recenter using aligned shifts: on

All other parameters: default settings

22. After the particles have been re-extracted, input them into another ***Non-uniform Refinement*** job using the volume from the previous ***Non-uniform Refinement*** job. Misaligned particles may still be present in the final volume after this step. Multiple rounds of ***3D classification*** and volume alignment may therefore be required to fully align all filament ends.

Symmetry: C1

All other parameters: default settings

23. Once actin filament ends have been realigned, weak density corresponding to the end-binding protein may become apparent. For CAP, weak density was observed bound to the D-loop of the two terminal pointed-end subunits, roughly consistent with the known structure of the helical folded domain (HFD) [20], whereas other CAP domains were absent (**Figure 1**, middle panel). For weaker end-binding proteins, populations of bound and unbound ends are often mixed. To improve occupancy, generate a mask corresponding only to the end-binding protein and use it in a ***3D Classification*** job, together with the particles from the final ***Non-uniform Refinement*** job. This typically yields separate volumes with and without the end-binding protein. Multiple rounds of 3D classification may be necessary to separate particles containing the end-binding protein.

Number of classes: 5

Filter resolution (Å): 6

All other parameters: default settings

24. Input classes containing the end-binding protein, along with the corresponding volumes, into a new ***Non-uniform Refinement*** job. Even if mixed populations of the end-binding protein are present within each class, it is generally preferable to separate these only after full occupancy of the protein has been achieved.

Symmetry: C1

All other parameters: default settings

25. Now that occupancy of the end-binding protein is improved, mixed populations corresponding to distinct conformations may become apparent. For CAP, one HFD was consistently observed bound to the terminal actin, while weak density for a second HFD was sometimes visible on the penultimate actin (**Figure 1**, bottom panel). To separate these populations, generate a mask around the weak density and use it in a ***3D Classification*** job.

Number of classes: 5

Filter resolution (Å): 6

All other parameters: default settings

*Note: The mask used for this step will depend on the occupancy of each state and on how the ABP binds to the filament end. It is advisable to test different masks at this stage to determine which one provides the best separation (masks encompassing only the mixed density, including actin subunits, the whole end, or unique regions may each be tested for separating different particle classes). In some cases, reusing the end-binding protein-specific mask from step D23 may be sufficient for class separation.*

26. Now, separate the different end-binding protein state classes into their own respective ***Non-uniform Refinement* jobs.** This will be the final refinement step before post-processing.

Symmetry: C1

Minimize over per-particle scale: on

All other parameters: default settings

27. For each filament end state, start a *Global CTF Refinement* job followed by a *Local CTF Refinement* job, and finally input the resulting particles into a *Non-uniform Refinement* job using the locally CTF-refined particles and the most recent volume from each final refinement. These jobs will correct for local changes in CTF that are not accounted for in the ***Patch CTF*** job done earlier. It is recommended to try correction for all parameters [spherical aberration, tetrafoil, anisotropic mag, and Ewald sphere (EWS) curvature] in case those parameters can improve resolution.

***Global CTF Refinement*** job:

Number of iterations: 2

Fit spherical aberration: on

Fit tetrafoil: on

Fit anisotropic mag.: on

Account for EWS curvature: on

All other parameters: default settings

*Note: Some of these parameters only improve high-resolution structures; however, it is still worth trying each one to see if it improves the resolution.*

***Local CTF Refinement*** job:

All parameters: default settings

***Non-uniform Refinement* job:**

Symmetry: C1

Minimize over per-particle scale: on

All other parameters: default settings

*Note: Global and local CTF refinement can be performed on the fly using their corresponding options in the* ***Non-uniform Refinement*** *job.*

28. Now, use these CTF-refined particles, volume, and original micrographs in a ***Reference-Based Motion Correction*** job, followed by a final ***Non-uniform Refinement*** job.

***Reference-Based Motion Correction*** job:

Number of GPUs: variable

All parameters: default settings

***Non-uniform refinement*** job:

Minimize over per-particle scale: on

Symmetry: C1

All parameters: default settings

29. The volumes are now final and can be validated in the next section.

**F. Validation of cryo-EM maps using common plots**

Now that the maps have been finalized, we must validate them using several metrics to make sure they are optimal. For this example, the Fourier shell correlation (FSC) curves, the orientation distribution plot, the local resolution, and the directional FSC or 3D FSC plots generated by cryoSPARC will be analyzed. Any of these plots can and should be used during data processing to assess map quality iteratively.

**Figure 4.**
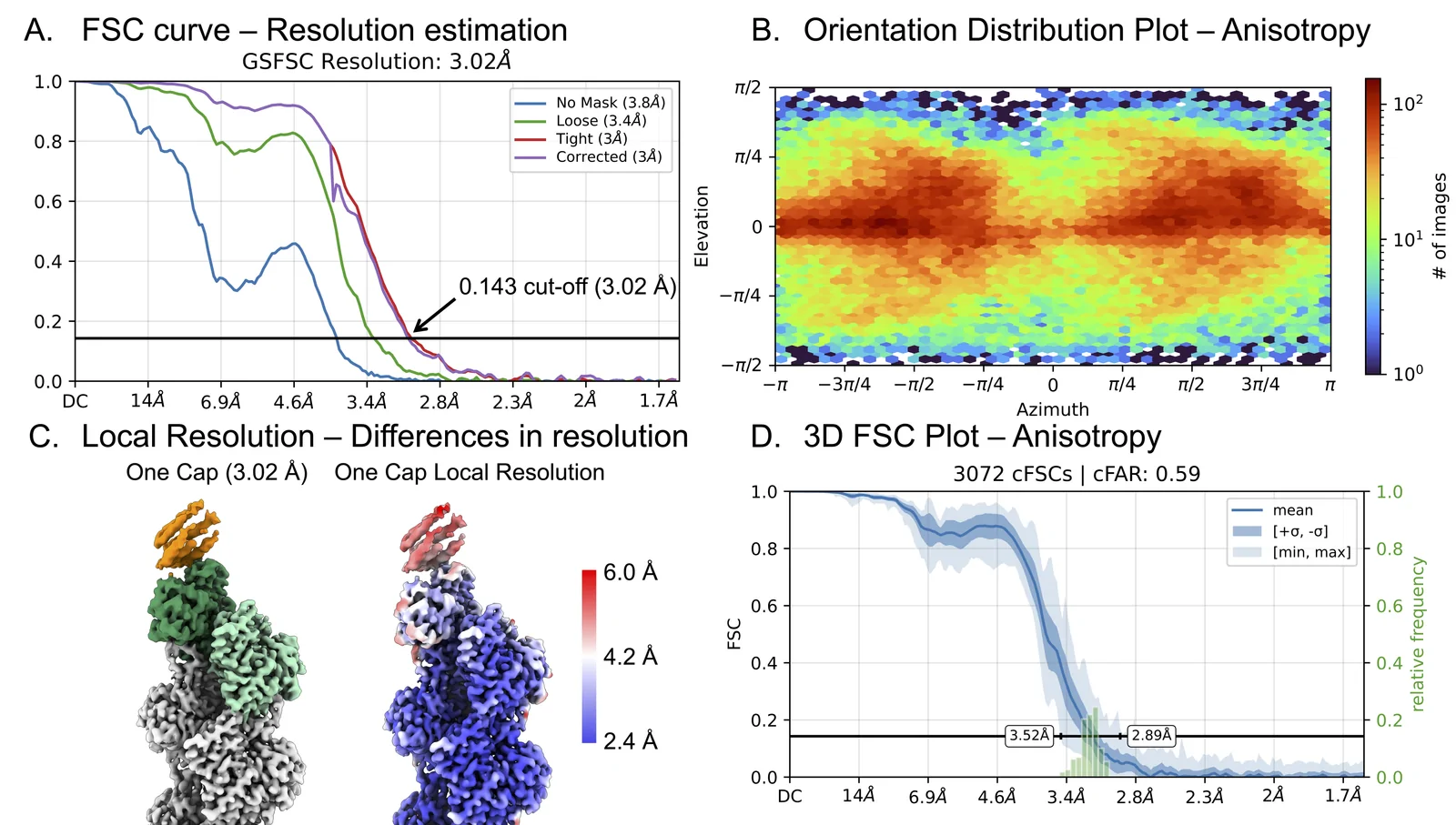
Example validation plots and figures from cryoSPARC. A) Fourier shell correlation (FSC) plot used to determine the resolution of a cryo-EM map. The gold standard 0.143 cutoff is highlighted and is the value at which the global resolution is derived. B) Orientation distribution plot outputted by a ***Non-uniform Refinement*** job, which shows a heat map of viewing orientations of a cryo-EM map. C) A cryo-EM map colored by protein chain next to the same map colored by local resolution. The end-binding protein CAP has a much lower resolution than actin, which is not apparent from just the global resolution. D) An example of a 3D FSC plot, which combines an FSC curve with measures of anisotropy for a given cryo-EM map.

1. The first metric that is often looked at with cryo-EM maps is their resolution, which is the resolution when the FSC curve is at 0.143, as shown in **Figure 4A**. This plot can be found in the event log of a cryoSPARC ***Non-uniform Refinement*** job. A common problem easily noticed by an FSC plot is that the curve never reaches zero, which artificially inflates the resolution. This is most likely due to duplicate particles and can be fixed easily using the ***Remove Duplicate Particles*** job on the particles from the reconstruction.

2. Cryo-EM maps are generated from the 2D projected images that are reconstructed into a 3D map. This map is said to be isotropic if there is a uniform resolution from all viewing orientations. If a map is anisotropic, it is likely caused by a preferred orientation of the particle during grid freezing or during data processing if the particle picking method is biased toward certain orientations. A viewing direction distribution plot can visualize the abundance of images from certain views of a cryo-EM map, as seen in **Figure 4B**. In this plot, there appears to be a bias in certain orientations; however, there remains full coverage along the azimuth. See the Troubleshooting section if it is suspected that orientation bias is from the particles themselves. If the issue is suspected to be with the particle picking ML model, returning to manual picking in E5 and manually picking another 1,000 particles can usually improve the Topaz model.

3. While the global resolution, as determined with the FSC curve, is useful, cryo-EM maps can have varying resolutions throughout the map. Often, for actin filament ends, the actin filament portion of the map has significantly higher resolution than the filament-binding protein. The primary way to assess this discrepancy is by using a ***Local Resolution Estimation*** job, which will allow you to visualize the local resolution of a given cryo-EM map in ChimeraX. An example filament end map colored by local resolution is shown in **Figure 4C.** Here, the global resolution is approximately 3 Å; however, this is mostly driven by the high resolution of the actin filament, while the CAP molecule has approximately 6 Å resolution. This is usually inherent to the way the end-binding protein binds, and more loosely bound particles generally have lower resolution.

4. A more quantitative tool other than the orientation distribution plot to assess anisotropy of a cryo-EM map is the 3D FSC plot, which can be found by running an ***Orientation Diagnostics*** job on the particles and volume from the final ***Non-uniform Refinement*** job. An example plot generated by this program for the example project can be seen in **Figure 4D**. The metric cFAR, listed on top, is a single quantitative metric that describes how isotropic maps are, with values closer to 1 being isotropic and values closer to 0 being anisotropic. A value of 0.59 for this map indicates that it is moderately isotropic but does have some orientation bias. The graph shows the range of cFSC curves from different orientations; the tighter this range, the more isotropic the map is in general. For more information on these types of plots, see [23].

## Validation of protocol

This protocol has been developed and applied by our laboratory in the determination of several published and unpublished filament end structures [11,13–15].

## General notes and troubleshooting

1. This protocol utilized Quantifoil R1.2/1.3 300 mesh grids for sample preparation; however, other grid types can be used as well. Most changes within a grid have tradeoffs between data quality and/or collection speed and the strength of the grid itself. The hole spacing pattern used here was a 1.2 μm hole size with 1.3 μM spacing between the holes. Other common grid spacing patterns increase the hole size to 2 μm, with 1–2 μm spacing between holes. The larger the hole size, and the closer the holes are together, the more imageable area there is for data collection, but the more fragile the grid is during sample preparation. The support mesh has similar trade-offs and most commonly ranges from 200 to 400, referring to the number of squares on a single grid. Lower-mesh grids (e.g., 200-mesh) have more holes per square, which may allow more images to be collected per session, but they are more susceptible to damage during freezing. In contrast, 400-mesh grids are stronger but provide less imageable area for data collection. R1.2/1.3 300-mesh grids were chosen for this project because they provide a balance between strength and imageable area.

2. Actin barbed ends polymerize more rapidly than pointed ends; therefore, it is generally easier to limit filament growth at the barbed end. For proteins that bind the pointed end, the capping protein (CP) can efficiently generate short filaments. In contrast, proteins that bind weakly or do not effectively block barbed-end elongation may require the use of mechanically sheared filaments, as was required for some formins [13].

3. Creating appropriate masks can be tricky and require multiple attempts and/or parameters chosen. If 3D classification does not seem to filter out particles, you may try making the mask using more or less area in ChimeraX or varying the dilation radius or other parameters in the ***Volume Tools*** job.

4. The data processing workflow is often not linear, and individual steps may need to be repeated. In addition, multiple masking strategies may be required to obtain the final structures.

5. This protocol may also be applicable to other filamentous protein systems, provided that filament length can be controlled such that a sufficient number of filament ends are present within grid holes.

**Troubleshooting**

**Problem 1:** Filament ends do not separate from filament middles during 3D classification.

Potential cause: The filament tips may be positioned too close to the edge of the box.

Potential solution: In cryoSPARC, use the ***Volume Alignment Tools*** job to shift all particles used as input for the ***3D classification*** job toward the barbed end. Re-extract these particles and perform a new ***3D classification***. Repeat the procedure for pointed-end particles. This typically yields a barbed-end class in the first classification and a pointed-end class in the second.

**Problem 2:** Topaz Extract job yields few particles.

Possible cause: The particle threshold value is too high.

Possible solution: In the ***Topaz Extract*** job, decrease the particle threshold by increments of 1 or more to increase particle picking. This will also increase the number of junk and mid-filament particles; however, these can be removed in later ***Heterogeneous refinement*** and ***3D classification*** steps.

**Problem 3:** Preferred orientation for final end structures.

Possible cause: Orientation distribution issues can be caused by numerous factors, but often, with filament end structures, the issue is related to particle interactions with the air–water interface.

Possible solution: There are many ways to reduce preferred orientations in cryo-EM samples; however, we have observed that different detergents can reduce this bias. The detergent NP40 has worked several times to improve orientation distribution, but several detergents should be tested for every new protein. Another solution is to collect cryo-EM images at a tilt, which can limit resolution but improve the overall quality of anisotropic maps.

**Problem 4:** It is too difficult to pick filament ends on noisy micrographs.

Potential cause: There could be many causes for noise in micrographs, usually related to data collection issues.

Potential solution: Using the ***Micrograph Denoiser*** job, you may be able to reduce the noise of micrographs effectively enough to pick particles. This job uses a machine learning–based approach to increase the signal-to-noise in micrographs. However, ***Micrograph Denoiser*** does not always help, and it would be best to improve the quality of data during collection.
